# “Things Have Changed”—Laparoscopic Cytoreduction for Advanced and Recurrent Ovarian Cancer: The Experience of a Referral Center on 108 Patients

**DOI:** 10.3390/cancers15245726

**Published:** 2023-12-06

**Authors:** Marcello Ceccaroni, Giovanni Roviglione, Francesco Bruni, Susan Dababou, Martina Venier, Carlotta Zorzi, Matteo Salgarello, Giacomo Ruffo, Filippo Alongi, Stefania Gori, Lorenza Driul, Stefano Uccella, Fabio Barra

**Affiliations:** 1 Department of Obstetrics and Gynecology, Gynecologic Oncology and Minimally-Invasive Pelvic Surgery, International School of Surgical Anatomy, IRCCS Sacro Cuore “Don Calabria” Hospital, Negrar, 37024 Verona, Italy; issaschool@gmail.com (M.C.); giovanni.roviglione@sacrocuore.it (G.R.); francesco.bruni@sacrocuore.it (F.B.); carlotta.zorzi@sacrocuore.it (C.Z.); 2 Department of Obstetrics and Gynecology, AOUI Verona, University of Verona, 37129 Verona, Italy; sdababou@gmail.com (S.D.); stefano.uccella@univr.it (S.U.); 3 Obstetrics and Gynecology, Department of Medical Area (DAME), University of Udine, 33100 Udine, Italy; martina04venier@gmail.com (M.V.); lorenza.driul@uniud.it (L.D.); 4 Department of Nuclear Medicine, IRCCS Sacro Cuore “Don Calabria” Hospital, Negrar, 37024 Verona, Italy; matteo.salgarello@sacrocuore.it; 5 Department of General Surgery, IRCSS “Sacro Cuore-Don Calabria” Hospital, Negrar di Valpolicella, 37024 Verona, Italy; giacomo.ruffo@sacrocuore.it; 6 Department of Advanced Radiation Oncology, IRCCS Sacro Cuore Don Calabria, 37024 Verona, Italy; filippo.alongi@sacrocuore.it; 7 Department of Medical Oncology, IRCCS Sacro Cuore “Don Calabria” Hospital, Negrar, 37024 Verona, Italy; stefania.gori@sacrocuore.it; 8 Department of Health Sciences (DISSAL), University of Genoa, 16132 Genoa, Italy

**Keywords:** epithelial ovarian cancer, laparoscopy, primary debulking surgery, secondary debulking surgery, minimally invasive surgery, optimal cytoreduction

## Abstract

**Simple Summary:**

Minimally invasive laparoscopic surgeries improve surgical recovery with shorter hospital stays and lower complications. However, the role of minimally invasive surgery in advanced and recurrent ovarian cancer has remained confined to small case series. This retrospective study reports the highest number of patients with advanced and recurrent ovarian cancers undergoing laparoscopic surgery in a single referral center. A rigorous algorithm for the selection of patients has been applied to confirm the feasibility of laparoscopy for primary debulking surgery and broadened its application to interval and secondary debulking surgery. Our study showed that minimally invasive surgery is feasible in select patients with favorable postoperative and oncological outcomes, consistent with other cohorts reported in the literature undergoing traditional laparotomic approach.

**Abstract:**

Objective: To report the feasibility of laparoscopic cytoreduction surgery for primary and recurrent ovarian cancer in a select group of patients. Methods: A retrospective analysis was conducted on a cohort of patients with FIGO stage IIIA-IV advanced ovarian cancer who underwent laparoscopic primary debulking surgery (PDS), interval debulking surgery (IDS), or secondary debulking surgery (SDS) between June 2008 and January 2020. The primary endpoint was achieving optimal cytoreduction, defined as residual tumor less than 1 cm. Secondary endpoints included evaluating surgical complications and long-term survival, assessed at three-month intervals during the initial two years and then every six months. Results: This study included a total of 108 patients, among whom, 40 underwent PDS, 44 underwent IDS, and 24 underwent SDS. Optimal cytoreduction rates were found to be 95.0%, 97.7%, and 95.8% for the PDS, ISD, and SDS groups, respectively. Early postoperative complications (<30 days from surgery) occurred in 19.2% of cases, with 7.4% of these cases requiring reintervention. One patient died following postoperative respiratory failure. Late postoperative complications (<30 days from surgery) occurred in 9.3% of cases, and they required surgical reintervention only in one case. After laparoscopic optimal cytoreduction with a median follow-up time of 25 months, the overall recurrence rates were 45.7%, 38.5%, and 39.3% for PDS, ISD, and SDS, respectively. The three-year overall survival rates were 84%, 66%, and 63%, respectively, while the three-year disease-free survival rates were 48%, 51%, and 71%, respectively. Conclusions: Laparoscopic cytoreduction surgery is feasible for advanced ovarian cancer in carefully selected patients, resulting in high rates of optimal cytoreduction, satisfactory peri-operative morbidity, and encouraging survival outcomes. Future studies should focus on establishing standardized selection criteria and conducting well-designed investigations to further refine patient selection and evaluate long-term outcomes.

## 1. Introduction

Ovarian cancer ranks as the seventh most common cancer and the eighth leading cause of tumor-related death in women [1]. Each year, it is estimated that 65,000 cases are diagnosed in Europe, including almost 5000 in Italy [2]. The majority of cases are diagnosed at an advanced stage, with a 5-year survival rate below 45% [3]. Debulking surgery through an open approach followed by platinum-based chemotherapy is the standard treatment approach for advanced ovarian cancer [4]. Optimal cytoreduction, defined as microscopic residual disease or a residual tumor (RT) less than 1 cm, is associated with better disease-free survival and overall survival rates [5]. Neoadjuvant chemotherapy followed by interval debulking surgery (IDS) has been investigated as an alternative treatment strategy in recent years, showing higher rates of optimal debulking without compromising survival compared with primary debulking surgery (PDS) [6,7]. However, the optimal treatment strategy between PDS and IDS is still under debate.

Traditional cytoreductive surgery for advanced ovarian cancer is typically performed through a xipho-pubic incision, associated with possible major complications and prolonged hospitalization [8]. Recent randomized studies have emphasized the importance of complete resection in recurrent ovarian cancer [9,10,11,12]. Minimally invasive laparoscopy has emerged as a promising alternative for early-stage ovarian cancer, offering advantages such as higher patient satisfaction, shorter hospital stays, faster recovery, and lower complication rates compared with laparotomy [13,14,15]. However, the role of minimally invasive surgery in advanced and recurrent ovarian cancer has been limited to small case series [8,16,17] or pre-operative diagnostic evaluations [18,19]. Laparoscopic IDS has shown promising results in terms of feasibility, morbidity, and survival rates [12,20,21]. As a result, laparoscopy has increasingly been considered for PDS, although experience in this area is still limited. The technical challenges associated with laparoscopic surgery, such as the inability to visualize and palpate the entire peritoneal surface and retroperitoneal structures, require proficient laparoscopic skills to achieve complete cytoreduction [8]. 

Our previous study demonstrated the feasibility and outcomes of laparoscopic PDS in a strictly selected group of patients with advanced ovarian cancer. Additionally, we provided data on patients who were not eligible for laparoscopy and underwent traditional open surgery. Although not a randomized comparison, this study highlighted the potential role of laparoscopic PDS in a well-selected patient population [8]. The current study aims to confirm the feasibility of laparoscopic PDS and expand its application to IDS and SDS for recurrent ovarian cancer, utilizing the same rigorous selection criteria.

## 2. Materials and Methods

This retrospective analysis utilized a prospectively collected database of patients who underwent laparoscopic debulking surgery for advanced or recurrent ovarian cancer at the Gynecologic Oncology Unit of the IRCCS “Sacro Cuore-Don Calabria” Hospital Negrar of Valpolicella (Verone, Italy). This study was conducted following ethical committee approval and reported according to the STROBE statement [22].

Patients underwent pre-operative evaluations, including anamnestic data collection, clinical examination, CA125 measurement. Pelvic ultrasound and whole-body positron emission tomography/computed tomography (PET/CT) scan were used for preoperative evaluation to assess tumor extension, resectability of tumor, and identification of metastatic disease. A multidisciplinary team consisting of gynecologic oncologic and general surgeons, oncologists, urologists, radiologists, and anesthesiologists reviewed each case. Patients with suspected FIGO (The International Federation of Gynecology and Obstetrics) stage III-IV primary or recurrent ovarian cancer and no contraindications to aggressive surgery underwent diagnostic laparoscopy to assess the feasibility of complete cytoreduction.

The selection criteria for PDS and IDS in initially diagnosed advanced ovarian cancer were based on the Fagotti Score, which assessed peritoneal carcinomatosis, diaphragmatic involvement, mesenteric involvement, omental involvement, small bowel involvement, stomach infiltration, and liver metastasis [23]. PDS was performed if the Fagotti score was <8 and the patient was deemed suitable for extensive surgery based on clinical and anesthesiologic considerations. Recurrent cases underwent imaging studies and diagnostic laparoscopy to determine the feasibility of SDS with optimal RT. In particular, the decision to undergo SDS was based on a comprehensive evaluation of the individual patient’s clinical condition, overall health status, previous treatments, and the extent of disease recurrence.

Subsequently, patients were evaluated to determine eligibility for laparoscopic or open abdominal approach for PDS, IDS, and SDS. Laparoscopic cytoreduction was not performed if at least one of the following exclusion criteria was present [8]: massive omental “cake”, obliteration of Morison’s pouch, necessity of more than two liver resections, posterior right or left diaphragmatic wall involvement, multi-visceral carcinomatosis, bulky upper abdominal mass > 5 cm, pelvic bulky disease > 20 cm, multiple diaphragmatic infiltrative carcinomatosis, bilateral diaphragmatic involvement, necessity of more than two bowel resections, necessity of more than one lymph-nodal debulking procedure, or obliterated spleno-colic ligament/angle/left diaphragm.

In the case of PDS, all patients received six cycles of adjuvant platinum-based combination chemotherapy. Newly diagnosed advanced ovarian cancer cases considered unsuitable for PDS underwent three or four cycles of platinum-based neoadjuvant chemotherapy followed by three cycles of adjuvant chemotherapy. Patients with recurrent ovarian cancer received adjuvant chemotherapy. Starting in 2013, bevacizumab was incorporated into the frontline or second-line treatment regimen. 

The primary endpoint of the study was to evaluate the efficacy of laparoscopic surgery in achieving optimal cytoreduction, defined as RT less than 1 cm, for advanced ovarian cancer. Secondary endpoints included surgical complications and long-term survival. Post-operative complications were described according to the Clavien–Dindo classification [24] and were categorized as “early” or “late” depending on whether they occurred within or after 30 days from surgery, respectively.

Follow-up assessments were conducted at regular intervals, with clinical evaluations and tumor marker measurements scheduled every three months for the first two years. Subsequently, evaluations were performed every six months for the following three years, followed by annual evaluations. Thoraco-abdominal CT scans were conducted before surgery, before initiating chemotherapy, and one month after completing adjuvant treatment. These CT scans were repeated annually. Abdominal ultrasound was performed annually as part of the follow-up protocol. PET/CT scans were conducted only when there was suspicion of recurrent disease [25].

### 2.1. Surgical Technique

The procedures were performed by the senior author (M.C.), who has extensive experience in surgical anatomy and gynecologic surgery (more than 2500 major gynecologic operations, with more than 500 gynecologic oncology cases).

All procedures were conducted under general anesthesia with the patient in a dorsal lithotomy position and legs secured in Allen stirrups. Trocar placement followed a standardized technique, with a 10 mm laparoscope inserted through the umbilical position and three 5 mm trocars placed at the suprapubic level, left iliac fossa, and right iliac fossa. Additional trocars were used as needed, such as a 5 mm trocar at the right hypochondrium for cases involving bowel segmental resection or a 12 mm trocar replacing the right iliac trocar. Procedures involving the upper abdomen eventually required an extra trocar in the upper right hypochondrium. Trocar placement and adjustments were customized for each case to ensure optimal access and visualization during laparoscopic cytoreduction.

The surgical procedure began with a thorough evaluation of peritoneal surfaces, recesses, and splanchnic organs using a high-definition video laparoscope. Standard operative procedures included total or radical hysterectomy, uni- or bilateral adnexectomy, infra or gastro-colic omentectomy, peritonectomy, and removal of visible and laparoscopically palpable metastatic lesions. Total extra-fascial hysterectomy followed the Clermont–Ferrand technique, while radical hysterectomy was subclassified according to Querleu–Morrow’s classification [26]. In cases of extensive pelvic infiltration of the peritoneum, posterior pelvic exenteration with Hudson-Delle Piane radical retrograde hysterectomy was performed, sometimes with en bloc rectal resection using a nerve-sparing technique [27]. Lymphadenectomy or dissection of pelvic and para-aortic nodes was carried out only when bulky nodes or suspicious retroperitoneal disease were identified. 

The laparoscopic dissection of the paraaortic retroperitoneum employed a transperitoneal approach, which involved opening the retroperitoneum at the root of the mesentery at the right promontorium and lifting the peritoneal tent up to the duodenum. For direct exploration and palpation of the paraaortic retroperitoneum and pelvic viscera, a hand-assisted laparoscopic approach was used. This involved a suprapubic mini-laparotomic transverse incision (<4 cm), allowing a hand to be inserted into the abdomen to palpate the retroperitoneum, omentum, and splanchnic viscera while maintaining laparoscopic vision. This procedure facilitated systematic exploration and identification of any millimetric residual disease under direct visual laparoscope guidance. The Alexis Laparoscopic System^®^ (Applied Medical, Amersfoort, The Netherlands) was employed to ensure exposure and prevent contamination of the abdominal wall when extracting specimens through the mini-laparotomic access. 

Additional abdominal procedures, such as bowel resection, bladder resection, liver resection, or diaphragmatic resection, were performed as necessary. Millimetric residual disease in the pelvis, abdominal peritoneum, intestinal mesentery, and diaphragm were destroyed using a laparoscopic 10 mm argon-beam coagulator. 

At the end of the procedure, RT was estimated. 

### 2.2. Statistical Analysis

Data were collected by using dedicated software (EGES software v.3.0.10; Mitcom, Mantua, Italy). Statistical analysis was performed using JMP 14.2.0 (SAS Institute Inc., Cary, NC, USA), GraphPad Prism 7.0 (GraphPad Software, San Diego, CA, USA), and SPSS Statistics 23.0 (IBM, Armonk, NY, USA). 

Survival analysis was performed by calculating median time-to-relapse, disease-free survival, and overall survival. Kaplan–Meyer curves and log-rank tests were estimated for disease-free survival and overall survival. Statistical significance was set at *p* < 0.05.

## 3. Results

### 3.1. Population

From June 2008 to January 2020, 135 patients were diagnosed with FIGO stage IIIA-IV ovarian cancer, with a mean (±SD) of 10.4 ± 6.6 patients with advanced ovarian cancer treated at our institution annually during the period considered.

Following a comprehensive evaluation of cytoreducibility, 49 (36.3%) patients were considered suitable candidates for PDS, 58 (43.0%) for IDS, and 28 (20.7%) for SDS. Subsequently, based on the laparoscopic cytoreducibility assessment, 40 (81.6%), 44 (75.9%), and 28 (85.7%) of these underwent laparoscopic PDS, IDS, and SDS. Therefore, during the time evaluated in the analysis, an average of 8.3 ± 5.7 and 2.1 ± 1.5 patients per year, respectively, were treated using the laparoscopic and laparotomic approaches. 

The median (range) operative time for PDS, IDS, and SDS was 272 (range, 120–585), 272 (range, 100–470), and 180 (range, 80–600) minutes, respectively. High-grade serous carcinoma was the most common histology observed in the three groups, accounting for 55.0%, 77.3%, and 80.0%, respectively. The other demographic and histologic characteristics of patients are listed in Table 1.

### 3.2. Surgical Results

Intraoperative characteristics of the three groups have been reported in Table 2. The peritoneum’s extensive pelvic infiltration required Hudson-Delle Piane radical retrograde hysterectomy in 67.5%, 68.2%, and 12.5% of patients in the PDS, IDS, and SDS groups, respectively. En bloc pelvic posterior exenteration was needed in 35.0%, 36.4%, and 12.5% of cases, respectively. About 50% of patients underwent upper abdominal surgery, such as atypical liver resection (Wedge resection), diaphragmatic surgery, Morison’s pouch peritonectomy, splenectomy, adrenalectomy, or cholecystectomy (Table 2). 

Optimal cytoreduction (RT ≤ 1) was achieved in 95.0%, 97.7%, and 95.8% of cases in the PDS, IDS, and SDS, respectively (with R0 of 87.5%, 88.6%, and 95.8%, respectively). Suboptimal cytoreduction (RT > 1) was registered in 5.0% (PDS), 2.3% (IDS), and 4.2% (SDS) of cases. Postoperative CT scans, which were performed in all the patients within 6 months of the surgical procedures, then confirmed these intra-operative measurements in all patients. In all these cases of suboptimal cytoreduction, it was deemed that even following conversion to a midline longitudinal laparotomy, achieving an optimal cytoreduction to al least RT < 1 cm would not have been feasible.

### 3.3. Surgical Complications

No intraoperative complications or conversions to open surgery were recorded. The median estimated blood loss was 200 mL (range, 25–1500) for PDS, 150 mL (range, 50–800) for IDS, and 100 mL (range, 50–800) for SDS.

Postoperative data, including early and late complications, can be found in Table 3. The median hospital stay was 9 days (range, 2–78). Early postoperative complications (n = 21; 19.2%) occurred in 20.0%, 20.5%, and 16.7% of cases following PDS, IDS, and SDS, respectively; these complications required a surgical reintervention within a month from previous surgery (n = 8) in 5.0%, 9.1%, and 8.3%, respectively. One patient who underwent IDS died due to respiratory failure immediately after surgery. Late postoperative complications (n = 10) were observed in 15.0%, 6.8%, and 4.2% of cases following PDS, IDS, and SDS, respectively (Table 3); a surgical reintervention after three months was required only in a patient who underwent IDS.

### 3.4. Oncological Outcomes

All patients included in the study were followed for at least six months, with a median time of 25 months (range, 6–115). Recurrence was diagnosed based on symptoms, imaging, or elevated CA125 levels. During the follow-up, the overall recurrence rate for patients with optimal cytoreduction was 45.7% (16/35) after PDS, 38.5% (15/39) after IDS, and 39.3% (9/23) after SDS. The most common sites of recurrence, in order of frequency, were the peritoneum (62.5%), retroperitoneal space (37.5%), and bowel (12.5%). Recurrent lesions in the abdominal wall were observed in only three cases (7.5%) with one port-site metastases (2.5%).

**Figure 1:**
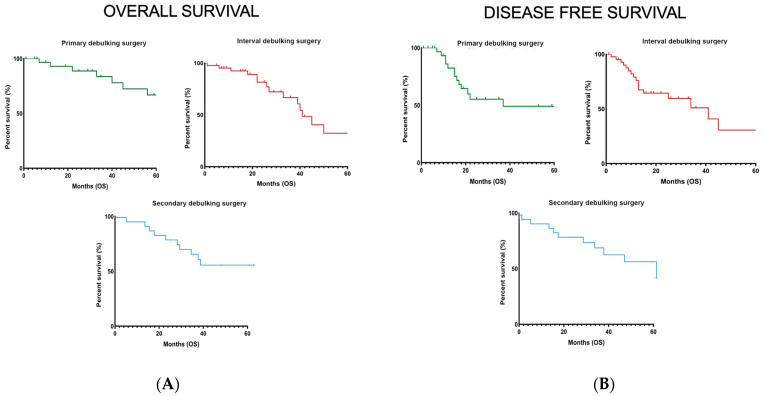
Kaplan–Meyer curves of overall survival (**A**) and disease-free survival (**B**) in the three groups of patients included in the study.

The median overall survival was 32 months (range, 1–115) after PDS, 35 months (range, 1–102) after IDS, and 22 months (range, 1–83) after SDS. Three- and five-year overall survival rates were 84% and 67% for PDS, respectively, 66% and 32% for IDS, and 63% and 58% for SDS, based on Kaplan–Meier analysis (Figure 1A). The median disease-free survival was 18 months (range, 7–104) after PDS, 7.5 months (range, 0.5–34) after IDS, and 7 months (range, 2–45) after SDS (Figure 1B). Three-year disease-free survival rates were 48%, 51%, and 71% for PDS, IDS, and SDS, respectively (Tables S1 and S2).

## 4. Discussion

This study provides the feasibility of laparoscopic surgery in advanced and recurrent ovarian cancer in a select patient group, with a high rate of perceived optimal cytoreduction and favorable long-term outcomes. Our data revealed a median overall survival of 32 and 35 months for PDS and IDS, respectively; large, randomized trials have previously reported median overall survival of 29 and 23 months for PDS and 30 and 24 months for IDS [7,28]. Therefore, our results align with previous data on traditional open-surgical approaches for advanced ovarian cancer (Table S3) [17]. Additionally, it has been reported that laparoscopy is a feasible option for SDS [16,29], for which the survival curves of our study were comparable to those described in the literature [20,30,31]. 

Over the past decade, advancements in technology, improved surgical skills, better pre-operative work-up, and increased focus on patient quality of life have made laparoscopy and minimally invasive surgery the preferred treatment for many gynecologic diseases [17,20,21,32]. However, the use of minimally invasive surgery in cancer has raised concerns about its impact on disease-free survival and overall survival. 

One of the important issues is related to accurately selecting patients who can be candidates for the minimally invasive approach. In this study, we initially employed the Fagotti score [23] to assess the probability of achieving optimal debulking surgery. If the score was less than 8 and the patient was deemed appropriate for extensive surgery based on clinical and anesthesiologic considerations, a PDS was undertaken. Subsequently, we applied rigorous criteria for selecting patients eligible for laparoscopic debulking surgery in advanced ovarian cancer, as outlined in our previous publication [8]. These criteria excluded patients with a large bulky upper abdominal mass and those requiring multiple lymph-nodal debulking procedures from consideration for laparoscopy. To address these contraindications, we incorporated preoperative PET/CT, known for its high accuracy in detecting lymph node metastatic disease and distant metastatic disease [33]. In this regard, it is crucial to acknowledge that the interpretation of the Fagotti score [23] and our laparoscopic criteria [8] involves subjective assessments, and the interpretation of certain criteria may vary among surgeons. Factors such as the extent of peritoneal carcinomatosis and the presence of residual disease can be open to interpretation, potentially introducing variability in scoring. Therefore, variations in surgical skill and proficiency may impact the reliability of these scores, whose applicability may be limited to specific surgical procedures or institutions, and their generalizability across diverse patient populations or surgical settings might be a point of consideration. Additionally, the score may not fully account for the influence of pre-operative imaging in assessing disease extent. Preoperative imaging modalities can provide valuable information, and their integration into the scoring system may enhance its accuracy. Lastly, while the Fagotti score has demonstrated utility in certain studies, the external validation of our laparoscopic criteria across multiple institutions [34,35,36] and patient cohorts is essential to establish their broader reliability and generalizability.

In our study, in cases of extensive pelvic infiltration of the peritoneum, posterior pelvic exenteration with a Hudson-Delle Piane radical retrograde hysterectomy was performed (more than 65% of cases belonging to PDS and IDS groups), sometimes with en bloc rectal resection using a nerve-sparing technique. This is because ovarian cancer is frequently diagnosed at advanced stages, leading to the obliteration of the Douglas pouch by the tumor and widespread pelvic peritoneal carcinomatosis. Radical surgical cytoreduction typically necessitates a retroperitoneal approach to pelvic masses, potentially involving multi-visceral resections in the upper abdomen. On this matter, the Hudson procedure remains a fundamental and benchmark technique for cytoreduction in fixed ovarian tumors. The establishment of avascular spaces is a crucial step in pelvic cytoreduction, providing additional access and facilitating the identification of anatomical structures in the retroperitoneum. Moreover, controlling pelvic hemorrhage becomes more manageable when all potential spaces near the surgical field are dissected. The development of Okabayashi’s pararectal space, the ligation of the uterine artery above the ureter, and the proximal transection of the lateral ligament of the rectum (in the case of a rectosigmoid anastomosis) contribute to preserving the patient’s pelvic innervation [37]. The noteworthy incidence of postoperative complications may be attributed to the radical nature of the cytoreductive surgery implemented in our cohort of patients.

Detractors of laparoscopy in advanced ovarian cancer have questioned its efficacy due to other technical limitations that may hinder optimal cytoreduction. These limitations include the difficulty in exploring specific abdominal and retroperitoneal organs, the potential peritoneal dissemination of tumor cells with carbon dioxide, and the risk of port-site metastases [38]. Nevertheless, we utilized the hand-assisted laparoscopic approach to palpate lower and upper abdominal organs at the end of the procedure, improving the ability to detect and remove any disease implant (and missing metastases) as well as enhancing the accuracy of residual tumor evaluation [39]. However, challenges in exploring the retroperitoneum, bowel, and mesentery remain areas of concern, despite no statistically significant difference in the rate of bowel and nodal recurrences between laparotomic and laparoscopic primary cytoreduction [8]. 

In our study, the median length of stay was nine days, similar to our previous findings, despite exclusively performing laparoscopic surgeries. Several factors contribute to this finding, including patient characteristics, surgical complexity, and postoperative care. Cases that necessitated extensive cytoreduction or, in a few instances, additional surgical interventions could have extended the recovery period, resulting in a longer length of hospital stay. A significant factor is that many patients referred to our center for advanced and recurrent ovarian cancer treatment come from distant locations. As a result, we need to ensure that they are in a stable condition with a low risk of complications before discharging them, following our hospital policy and ensuring their safety and well-being. 

This study is the largest series of patients with advanced and recurrent ovarian cancer undergoing laparoscopic surgery in a single center. As this is a single-surgeon, single-institution study, the evidence related to the results of this study is limited and cannot be generalized. The impact of a learning curve and modifications in surgical outcomes over time should be considered. As the study spanned a considerable timeframe, it is reasonable to assume that the accumulating experience and potential refinements in surgical techniques may have influenced the results. While a formal assessment of the learning curve was not performed in this study, we acknowledge that the surgeon’s experience and evolving expertise may have played a role in surgical outcomes. Additionally, the evolving landscape of surgical experience, advancements in minimally invasive instruments, as well as changes in perioperative and postoperative management over the study duration, should be also acknowledged as potential limiting factors in the interpretation of data. Another weak point of the study is the absence of a comparison group for open surgery to compare long and short-term peri-operative outcomes and complications. Lastly, although our study included patients with different disease settings, including upfront, interval, and secondary surgery, we recognize the future importance of analyzing these settings separately to better understand their unique outcomes and implications. 

Our retrospective analysis provides valuable insights into the feasibility of laparoscopic debulking surgery in centers dedicated to minimally invasive procedures by expert operators. Nevertheless, the inherent limitations related to the extended study duration and the dynamic nature of surgical practices underscore the need for further prospective investigations with more defined parameters. Such studies would offer a clearer understanding of the impact of evolving surgical techniques and perioperative practices on the outcomes of advanced ovarian cancer patients undergoing laparoscopic procedures. 

## 5. Conclusions

This study confirms the feasibility of laparoscopy for PDS and extends its application to IDS and SDS in a strictly selected group of patients with advanced ovarian cancer, particularly in centers led by surgeons with significant expertise in minimally invasive procedures. Further prospective research is essential to establish reproducible selection criteria and enhance the understanding of the role of minimally invasive surgery in managing advanced ovarian cancer.

## Figures and Tables

**Table 1:** Demographic characteristics of study population.

	PDS (n = 40)	IDS (n = 44)	SDS (n = 24)
Age (yrs) [min–max]	55 [34–85]	64 [41–85]	59 [39–78]
Median BMI [min–max]	23.6 [17–30]	22.7 [15–33]	23.3 [16–40]
Histology (n, %)			
*Serous*	22 (55.0%)	34 (77.3%)	19 (80.0%)
*Undifferentiated*	10 (25.0%)	9 (20.5%)	5 (20.0%)
*Clear cells*	0	0	0
*Endometroid*	2 (5.0%)	0	0
*Mucinous*	1 (2.5%)	0	0
*Others*	5 (12.5%)	1 (2.3%)	0
FIGO Stage (n, %) *			
*IIIA*	4 (10.0%)	0	3 (12.5%)
*IIIB*	6 (15.0%)	2 (4.7%)	4 (16.7%)
*IIIC*	26 (65.0%)	30 (69.8%)	13 (54.2%)
*IV*	4 (10.0%)	12 (27.9%)	4 (16.7%)

* for patients undergoing SDS, FIGO stage refers to the stage assigned at the initial diagnosis. PDS: Primary Debulking Surgery; IDS: Interval Debulking Surgery; SDS: Secondary Debulking Surgery; BMI: Body Mass Index.

**Table 2:** Intraoperative surgical data.

	PDS (n = 40)	IDS (n = 44)	SDS (n = 24)
Ascites (n, %)	9 (22.5%)	3 (6.8%)	0
EBL (mL) (median, min-max)	200 (25–1500)	150 (50–800)	100 (50–800)
EBL > 500 mL (n, %)	6 (15.0%)	5 (11.4%)	1 (4.3%)
OR time (min) (median, min-max)	272 (120–585)	272 (100–470)	180 (80–600)
**Specific surgical procedures (n, %)**			
No hysterectomy (previous)	2 (5.0%)	4 (9.1%)	14 (58.3%)
Hudson-Delle Piane	27 (67.5%)	30 (68.2%)	3 (12.5%)
Radical hysterectomy (Piver 2–3)	22 (55.0%)	34 (77.3%)	3 (12.5%)
Extrafascial hysterectomy	16 (40.0%)	6 (13.6%)	6 (25.0%)
Adnexectomy	38 (95.0%)	38 (88.4%)	5 (20.8%)
No adnexectomy (previous)	3 (7.5%)	6 (13.6%)	16 (66.7%)
Infracolic omentectomy	34 (85.0%)	28 (63.6%)	4 (16.7%)
Gastrocolic omentectomy	6 (15.0%)	14 (31.8%)	0
Systematic pelvic lymphadenectomy	6 (15.0%)	4 (9.1%)	2 (8.3%)
Systematic para-aortic lymphadenectomy	5 (12.5%)	0	0
Lymph node pelvic debulking	3 (7.5%)	3 (6.8%)	2 (8.3%)
Lymph node para-aortic debulking	5 (12.5%)	2 (4.5%)	1 (4.2%)
Appendectomy	9 (22.5%)	9 (20.5%)	0
Pelvic peritonectomy	24 (60.0%)	24 (54.5%)	5 (20.8%)
Colon resection	23 (57.5%)	23 (52.3%)	12 (50.0%)
Total colectomy	1 (2.5%)	0	0
Ileostomy (temporary)	9 (22.5%)	12 (27.3%)	2 (8.3%)
En-Bloc posterior pelvectomy	14 (35.0%)	16 (36.4%)	3 (12.5%)
Ileal resection	3 (7.5%)	5 (11.4%)	0
Double bowel resection	2 (5.0%)	4 (9.1%)	0
Atypical liver resection	4 (10.0%)	3 (6.8%)	2 (8.3%)
Glisson’s capsule stripping	1 (2.5%)	1 (2.3%)	1 (4.2%)
Glisson’s capsule argon laser	1 (2.5%)	2 (4.5%)	1 (4.2%)
Diaphragmatic stripping	13 (32.5%)	10 (22.7%)	5 (17%)
Diaphragmatic argon laser	4 (10.0%)	13 (29.5%)	0
Diaphragmatic resection	0	1 (2.3%)	0
Hepatic round/falciform ligament excision	3 (7.5%)	8 (18%)	0
Morrison’s pouch peritonectomy	3 (7.5%)	6 (13.6%)	2 (8.3%)
Splenectomy	0	0	1 (4.2%)
Cholecystectomy	0	0	1 (4.2%)
Adrenalectomy	0	0	1 (4.2%)
Bladder resection	2 (5.0%)	3 (6.8%)	0
Bladder shaving	2 (5.0%)	3 (6.8%)	0
Prevesical peritonectomy	20 (50.0%)	21 (47.7%)	2 (8.3%)
Ureteral stent	6 (15.0%)	7 (15.9%)	3 (12.5%)
Ureteroneocystostomy	1 (2.5%)	1 (2.3%)	2 (8.3%)
Abdominal wall mass debulking	4 (10.0%)	5 (11.4%)	1 (4.2%)
Vascular resection (epigastric vassels on mass of the abdominal wall)	0	1 (2.3%)	0
Total number of upper-abdomen surgery	19 (47.5%)	25 (56%)	10 (41.7%)

EBL: Estimated Blood Loss; OT: Operation Time; PDS: Primary Debulking Surgery; IDS: Interval Debulking Surgery; SDS: Secondary Debulking Surgery.

**Table 3:** Postoperative data.

	PDS (n = 40)		IDS (n = 44)		SDS (n = 24)	
	**CD Grade**	**Tot**	**CD Grade**	**Tot**	**CD Grade**	**Tot**
**Early complications (<30 days) (n, %)**						
Fever-Sepsis	CD I: 3	3 (7.5%)	CD I: 4 CD IIIa: 1	5 (11.4%)		-
Pleural effusion	CD I: 1 CD II: 1	2 (5.0%)		-		-
Polmunary Embolism		-	CD V: 1	1		-
Heart failure	CD II: 1	1 (2.5%)		-		-
Anemization	CD II: 2	2 (5.0%)	CD II: 1	1 (2.3%)	CD II: 1	1 (4.2%)
Monolateral hydroneophrosis		-		-		-
Ureteral fistula		-	CD IIIa: 1 CD IIIb: 1	2 (4.5%)	CD IIIb: 1	1 (4.2%)
Vaginal cuff dehiscence		-	CD IIIa: 1	1 (2.3%)		-
Recto vaginal fistula		-	CD IIIb: 1	1 (2.3%)	CD IIIb: 1	1 (4.2%)
Bleeding of rectal anastomosis	CD IIIa: 1	1 (2.5%)		-		-
Bowel obstruction	CD IIIb: 2 CD IV: 1	3 (7.5%)	CD IIIb: 2	2 (4.5%)		0
**Late complications (>30 days) (n, %)**						
Monolateral hydroneophrosis (ureteral stent)	CD IIIa: 3	3 (7.5%)	CD IIIa: 2	2 (4.5%)		-
Vaginal cuff dehiscence	CD IIIa: 2	2 (5.0%)		-		-
Rectalvaginal fistula	CD IIIa: 1	1 (2.5%)	CD IIIb: 1	1 (2.3%)		-
Hernia (laparocele)	CD I: 1	1 (2.5%)		-	CD I: 1	1 (4.2%)
Lymphocele	CD I: 1	1 (2.5%)		-		-

CD: Clavien–Dindo; PDS: Primary Debulking Surgery; IDS: Interval Debulking Surgery; SDS: Secondary Debulking Surgery.

## Data Availability

The data presented in this study are available on request from the corresponding author. The data are not publicly available due to hospital policy.

## Supplementary Materials

The following supporting information can be downloaded at: https://www.mdpi.com/article/10.3390/cancers15245726/s1, Table S1: Overall survival after surgical approaches for ovarian cancer; Table S2: Disease-free survival after surgical approaches for ovarian cancer; Table S3: Main studies on laparoscopic/laparotomic surgeries for advanced ovarian cancer.
