# Supplementary material for: “Things Have Changed”—Laparoscopic Cytoreduction for Advanced and Recurrent Ovarian Cancer: The Experience of a Referral Center on 108 Patients
Source: Cancers (Basel). 2023 Dec 6;15(24):5726. doi: 10.3390/cancers15245726 (PMC10741886; doi:10.3390/cancers15245726)
Supplement: Supplementary file 1 [file cancers-15-05726-s001.zip › cancers-2730836-supplementary.pdf]

Supplemental material

Supplementary Table 1. Overall survival after surgical approaches for ovarian cancer

SURGERY	OVERALL SURVIVAL					
	6 months	1 years	2 years	3 years	4 years	5 years
PDS	100%	93%	89%	84%	73%	67%
IDS	95%	92%	82%	66%	40%	32%
SDS	96%	96%	80%	63%	58%	58%

PDS: Primary Debulking Surgery; IDS: Interval Debulking Surgery; SDS: Secondary Debulking Surgery

Supplementary Table 2. Disease-free survival after surgical approaches for ovarian cancer

SURGERY	DISEASE FREE SURVIVAL					
	6 months	1 years	2 years	3 years	4 years	5 years
PDS	100%	86%	54%	48%	48%	40%
IDS	93%	76%	64%	51%	30%	30%
SDS	96%	92%	80%	71%	58%	54%

PDS: Primary Debulking Surgery; IDS: Interval Debulking Surgery; SDS: Secondary Debulking Surgery

Supplementary Table 3. Main studies on laparoscopic/laparotomic surgeries for advanced ovarian cancer

Authors, year	Study	Surgery		Pts (n)	FIGO stage	Residual Tumor (%, (RT))	Estimated blood loss (mL)	Complications IO (%)	Complications PO (%)	OS	PFS
Ceccaroni, 2018 [7]	Prospective	LPS	PDS	21	IIIA1/IV	95.3 (=0)	250	0.0	19	47.3 mo	42.3 mo
		LPT		45		88.4 (=0)	650	17.8	46.7	52.3 mo	45.2 mo
Liang, 2017 [30]	Retrospective	LPS	PDS	64	II/III/IV	85.9 (=0)	232	4.7	7.8	88.7% of pts at 3 years	55.5% of pts at 3 years
		LPT		68		67.6 (=0)	770	5.9	16.2	83.7% of pts at 3 years	33.3% of pts at 3 years
Melamed, 2017 [31]	Retrospective	LPS	IDS	450	IIIC/IV	46.9 (=0)	-	-	-	33.8 mo	-
		LPT		2621		49.5 (=0)	-	-	-	37.6 mo	-
Gueli Alletti, 2016 [20]	Retrospective	LPS	IDS	30	advanced ovarian cancer	96.6 (=0)	100	-	0	-	18mo
		LPT		65		95.4 (=0)	200	-	2 cases	-	12mo
Nezhat, 2012 [21]	Retrospective	LPS	SDS	19	I/II/III	78.9 (<1cm)	50	0	7.1	-	74.7% of pts at 6 mo

LPS: laparoscopy; LPT: laparotomy; PDS: primary debulking surgery; IDS: interval debulking surgery; IO: intraoperative; PO: postoperative; OS: overall survival; PFS: progression-free survival; mo: months; SDS: Secondary Debulking Surgery; pts: patients
